# Sleep in marathon and ultramarathon runners: a brief narrative review

**DOI:** 10.3389/fneur.2023.1217788

**Published:** 2023-09-22

**Authors:** Pantelis T. Nikolaidis, Katja Weiss, Beat Knechtle, Georgia Trakada

**Affiliations:** ^1^School of Health and Caring Sciences, University of West Attica, Athens, Greece; ^2^Institute of Primary Care, University of Zurich, Zürich, Switzerland; ^3^Medbase St. Gallen Am Vadianplatz, St. Gallen, Switzerland; ^4^Medical School, University of Athens, Athens, Greece

**Keywords:** exercise, athletic performance, marathon running, sleep deprivation disorders, sleep extension duration

## Abstract

**Introduction:**

Sleep is considered a fundamental biological function in humans necessary for recovery from daily physical activities. Considering the increasing popularity of long-distance running and participation in races such as marathons and ultramarathons, the aim of the present study was to review the relationship of such strenuous physical activities with sleep.

**Methods:**

A search of Scopus was performed on 24/6/2023 using the syntax [ABS (sleep) AND ABS (marathon)] to identify relevant papers, the references of which were hand-searched to find additional sources.

**Results:**

Optimal sleep has been shown to affect injury prevention and susceptibility to infection positively. In turn, participation in a marathon race may influence nocturnal autonomic modulation and disturb homeostasis. Ultramarathon races may have such a long duration that results in sleep deprivation even for several days, where sleep duration is quite below the physiological range. It seems that for ultramarathons of short duration, continuous running and sleep deprivation are beneficial for performance. In contrast, for races longer than 200 miles, it is necessary to develop sleep strategies to sustain performance.

**Conclusion:**

In summary, the longer the distance of a running race, the greater the importance of an optimal sleep for race performance as well as the impact of a race on sleep.

## Introduction

Sleep is considered a fundamental biological function in humans necessary for the recovery of energy loss resulting from daily physical activities including exercise and sports (1). Driller et al. (2) highlighted the importance of sleep in an athlete’s recovery process and performance, which may explain the increase in sleep monitoring in sports. In the context of daily energy expenditure, sport practice has been a subcategory of physical activity characterized by regular exercise training and often high metabolic demands (3). Sleep has been widely acknowledged as one of the foundations of sports performance, considering its impact on illness, injury, metabolism, cognition, memory, learning, and mood (4, 5). An explanation of the beneficial role of sleep on performance might be the restorative function of slow-wave sleep, allowing recovery from previous wakefulness and fatigue (4). Considering the increasing popularity of long-distance running and participation in races such as marathons and ultramarathons (6, 7), it would be interesting to examine the relationship of such strenuous physical activities with sleep. Therefore, the aim of the present study was to review sleep aspects of marathon and ultramarathon runners. For the purpose of our brief narrative review, a search of Scopus was performed on 24/6/2023 using the syntax [ABS (sleep) AND ABS (marathon)] to identify relevant papers, the references of which were hand-searched to find additional sources. The search did not have any time restriction resulting in 42 entries. The references of these studies were hand-searched to identify further literature. We considered the impact of sleep on marathon/ultramarathon performance and vice versa. First, the role of sleep in athletes generally was introduced, and second, thereafter, there was a focus on marathon and ultramarathon runners.

## Sleep and athletes

The model of sleep deprivation or partial sleep loss, observed in athletes traveling crossing several time zones or participating in prolonged races such as ultramarathons and triathlon races, was used as a methodological approach to examine the role of sleep (8). Compared with the general population, few studies have been conducted on the effects of sleep deprivation on athletes (9). Compared to the general population, elite athletes have poor quality and quantity of sleep due to training times, competition stress/anxiety, muscle soreness, caffeine use, and travel (4). There were several factors—including changes in nutrition, environmental aspects (e.g., temperature and altitude), crossing time zones and stress—that may exert detrimental effect on sleep, and consequently, on sports performance (10).

The beneficial role of exercise on sleep duration and delayed rapid-eye movement (REM) sleep onset, enhanced slow-wave sleep, and decreased REM sleep has been previously reported (11). Driver and Taylor (11) addressed methodological concerns about research on exercise and sleep with regard to differences in the study design (e.g., exercise protocols) and interactions between individual characteristics. Occasionally, athletes may be in a state of low energy availability (e.g., sudden decrease of energy intake in case of weight loss). A case study reported that weight loss practices did not compromise the sleep of martial sports athletes (12). Research on sleep patterns of athletes during the early coronavirus disease lockdown showed that sleep quality and quantity were characterized as “normal” for half of them, followed by those reporting “improved” and “worsened” sleep (13). In turn, sleep quality and quantity may relate to variability in sleep onset and offset (14) assessed by the sleep regularity index (SRI). A comparative study showed that SRI recorded for 5 days in athletes was better in competitive athletes, women, and athletes of individual sports (15). Driller et al. (16) found in a comparative study that individual-sport (e.g., badminton, boxing, cycling and rowing) athletes had greater total sleep time and higher sleep efficiency than team-sport (e.g., basketball, soccer, cricket, and hockey) athletes. In another study, SRI was monitored for 7 days and suggested that—compared to irregular sleepers—regular sleepers had greater sleep efficiency, less variability in total sleep time and sleep efficiency, similar total sleep time, and less variation in sleep onset times (14).

The feeling of stress before a sports competition may affect sleep. For instance, Vitale et al. monitored two nights before and two nights after an evening soccer game, and concluded that the athletes had a late bedtime and wake-up time after a game, whereas no alteration in sleep quality and duration was shown (17). Nevertheless, adolescent basketball players’ sleep quality did not differ in the competitive period compared to off-season (18).

In adolescent basketball players, the majority had sleep duration less than the suggested 8 h (19). Gupta et al. (20) reviewed 37 studies that showed an increased number of sleep complications in high-level athletes, and sleep disturbances were due to training, travel, and competition. They also highlighted the high incidence of insomnia symptoms such as increased sleep latencies, larger sleep fragmentation, non-restorative sleep, and disproportionate daytime fatigue. Regarding the role of traveling across several time zones, Fowler et al. (21) recorded sleep in physically active men who traveled from Australia (East) to Qatar (West) and vice versa and observed that sleep onset and offset occurred in a later time, and resting in bed as well as total sleep time were decreased during 4 days in Australia compared with baseline and Qatar.

## Sleep in marathon and ultramarathon runners

### Marathon

A methodological approach to examine the relationship between sleep and marathon was to investigate (a) the effect of different levels of sleep quality and quantity on marathoner runners’ physiology (e.g., immunity, musculoskeletal system), and (b) the effect of a marathon race on sleep quality and quantity. With regards to the relationship of sleep and immunity system in marathon runners, there has been a concern that strenuous and lengthy training or competing in endurance events (e.g., marathon races) may result in a high incidence of infections (e.g., upper respiratory tract infections) (22). For instance, Sparling et al. (23) reviewed selected scientific aspects of marathon running, and found that adequate sleep—in addition to proper nutrition, rest between intense training sessions, and the control of contact with sick people—may reduce susceptibility to infection. In addition to the immunity system, another health concern of marathon runners was the occurrence of musculoskeletal injuries. Marathon runners were subject to injuries, mostly in the lower limbs, related to overuse (24). Ashcroft (25) highlighted the role of adequate sleep in the context of the prevention of injuries in distance runners. In summary, there was evidence of a beneficial role of sleep for the immunity (infections) and musculoskeletal system (injuries).

The effect of a marathon race on sleep has been examined in studies that compared the condition of the race with training and rest (26–28). Montgomery et al. (26) investigated the effect of three circumstances (no exercise, a 90 min running, and a marathon race) on the sleep of recreational marathon runners. They showed a sleep disorder, consisted of an inhibition of REM sleep and a reduced in sleep duration after the marathon race, whereas sleep was unaffected by the 90 min training running. These authors attributed the sleep disruption after the marathon race to stress, as noted by the elevated cortisol levels (26). A similar study design (i.e., comparison of marathon race with training and rest) was adopted by Hynynen et al. (28) who studied the influence of a rest day, moderate endurance exercise, and marathon run on healthy, physically active men’s nocturnal heart rate (HR) variability. They observed that compared to a rest day, the intervals between consecutive heart beats increased to 109 and 130% after moderate endurance exercise and a marathon, respectively, whereas the standard deviation of these intervals decreased to 90 and 64%. These findings proposed a prolonged dose–response effect on autonomic modulation after exercises influencing nocturnal autonomic modulation and causing disturbance to homeostasis (28). Moreover, Cecchetti et al. (27) compared sleep HR after a habitual training session and a marathon race, separated by 2 weeks in senior marathon runners. They showed that HR was higher during marathon and post-marathon waking time than on the training day; however, no difference was found in sleep HR. The authors attribute this pattern to the decrease of adrenergic activation during night sleep (27). In summary, a marathon race likely would induce more sleep disturbances than a training session or no exercise; however, more research was needed in this field considering controversial findings (26–28).

Another aspect of practical significance was the traveling across time zones to participate in a marathon race (29). As it has been shown recently, a small number of participants in marathon races was from other countries and continents, and this observation concerned both elite and recreational runners (7, 30). For instance, from 1970 to 2017 in the New York City Marathon, ~530,000 out of ~1.2 M participants were not from USA (7). Traveling to the city of a marathon race might involve covering a large distance in a short time by plane resulting in circadian change and sleep loss (31). Moreover, sleep loss would also result from the spring version of daylight savings transition (DST), i.e., setting the clock 1 h forward (32), and might impair performance in case of a race in the day after a spring-DST (33). In this topic, O’Connor and Kancheva (33) examined the effect of DST on endurance performance and observed that the spring-DST marathon race time was slower by ~12 min while autumn-DST was slower by ~1 min compared to control marathon times. The authors proposed that their results indicate a deterioration of marathon running occurred on the spring-DST, which was attributed to a forced circadian change and sleep loss (33).

### Ultramarathon

It should be noted that a marathon race referred to running a distance of 42 km, whereas an ultramarathon race denoted any distance longer than 42 km or lasting more than 6 h (34). Thus, by definition, an ultramarathon race might cover a wide range of distances and durations (35), and the characteristics of ultramarathon runners might differ from marathon runners in terms of participation rates (e.g., fewer participants in ultramarathon than in marathon races) and performance (e.g., slower running speed in the former than in the latter races) (35). The effect of a race on sleep has been studied in ultramarathon races (36, 37). Bianchi et al. (36) examined the sleep–wake behavior of 200-mile (~82.5 h) ultramarathon runners before (for 7 days), during, and after a race (for 7 days). They reported that runners had ~5 h of sleep from ~5 sleep episodes (i.e., ~1 h of sleep per episode), and the sleep duration was 6.0 h before the ultramarathon, and 6.3 h in the week after the race. The authors concluded that runners drastically restricted their sleep, the importance of sleep increased during the days of the race, and the ultramarathoners had less sleep duration than the suggested ~8 h in both pre-race and post-race periods of the race (36). In another study of the same research group, Miller et al. (37) investigated sleep/wake behavior before, during, and after ultramarathon races lasting more than 161 km. The majority of runners of such distances usually had no sleeping during races, whereas, for races longer than 322 km, runners had more sleep episodes, more sleep time per episode and in total than races of 161–240 km. The authors concluded that for events lasting less than 161 km, the advantage of constant running values more than the disadvantage of unceasing wakefulness/sleep deprivation. On the contrary, for events lasting more than 322 km, there is an apparent compromise between sleep deprivation and race strategy, whereby runners may not tolerate an intended performance without sleep (37). In addition, they attributed this finding to the conventional sleep/wake behavior models indicating that sleep requirement increases as wakefulness increases, or in this case, as race duration increases. To sum up, ultramarathon runners sleep inadequately even in pre-and post-race periods, sleep duration decreases during race days, and the longer the race distance, the lower the sleep duration (36, 37).

Graham et al. (38) examined sleep during a 120-mile, three-day Arctic ultramarathon. Sleep—assessed by the Brunel Mood Scale questionnaire—was 4.07 h per day and correlated neither with injury rate nor mood changes. The authors interpreted these findings as suggesting that this demanding race involves significant psychological and physiological preparation that minimizes the effects of sleep deprivation (38). In another research, Huang et al. (39) studied the aspect of visual hallucinations commonly reported by adventure-race competitors (e.g., ultramarathons in the mountains and deserts) in a 245 km race with an altitude difference of 3,266 m (duration 44 h). All eight runners in this study slept for <30 min during the race and three had visual hallucinations, which the authors assumed may be associated with excessive physical exertion and sleep deprivation. Sleep deprivation may influence ultramarathon performance within a holistic model, including environmental conditions, painkillers or psychostimulants, and cognitive and nutritional strategies (40). During ultramarathon training and competing, to sustain performance and offset the compromise of athlete safety due to sleep deprivation, a consensus supports the strategic use of caffeine (41). Poussel et al. (42) observed that all runners in the North-Face UltraTrail du Mont-Blanc 2013 adopted pre-race sleep management strategies, the majority of runners did not sleep during the race and non-sleepers were faster than sleepers. In addition, ultramarathon finishers of the abovementioned race, who used a sleep management strategy based on increased sleep time before the race, were faster than those who did not use such strategy.

### Other aspects

In addition to the effect of marathon and ultramarathon races on sleep, a few studies examined the role of relevant aspects such as sleeping after traveling across different time zones (43), preparation for an endurance race (44), night running (45), post-marathon recovery (46), and sex differences (47). Montaruli et al. (43) studied the effect of a flight across different time zones (from Milan to New York) on the sleep of marathon runners divided into the morning training group, evening training group, and control group. Sleep patterns were continuously monitored using an actometer on the wrist of the non-dominant hand. They concluded that physical activity could positively affect sleep by improving quality and encouraging re-synchronization after the flight (43). In addition, Meijer et al. (44) examined the effect of a 20 week program on sleeping metabolic rate in athletes preparing for a half-marathon, where they measured sleeping metabolic rate from 3 to 6 am in a respiration chamber. No changes in SMR were found, either in absolute terms or when normalized for body mass or fat-free mass, concluding that exercise training has no chronic, long-term effect on sleeping metabolic rate (44). Furthermore, Rozmiarek et al. (45) contacted research in night runners and observed that night running makes it easier to fall asleep and improves the quality of sleep. However, Castell (48) proposed that in endurance athletes, one night’s sleep loss would induce changes in parameters related to immune function and cognitive ability. Polak et al. (46) analyzed Rotterdam Marathon runners for information concerning total recovery and recovery from pain, stiffness, loss of appetite, sleep disturbance and fatigue. They showed that the immediate replacement of 2.5 L of fluid had no significant influence on the total recovery rate, the number of days with pain or stiffness, the appetite, sleep, or fatigue (46). With regard to sex differences, Roberts et al. (47) studied endurance athletes in pre-race relationships between sleep, perceived stress and recovery. They monitored sleep using actigraphy over four consecutive days before an ultramarathon and showed that—compared with men—women had shorter wake after sleep onset (50 vs. 65 min), and experienced greater pre-race stress, and their sleep duration was associated with emotional factors (47). Swain and Rosencrance (49) focused on headaches in half-marathon and 5 km runners. They observed a higher proportion of distance runners with migraine headaches compared to the regular population (36% vs. 17%). An interesting finding was that running reduced the severity and frequency of all types of headaches, and sleep eliminated headaches in most headache patients (49). It was acknowledged that being a narrative review the present study could not draw quantitative conclusions. As the body of relevant literature increases, systematic reviews and meta-analyses are expected in the near future to provide complimentary knowledge.

## Conclusion

In summary, optimal sleep has been shown to positively affect injury prevention and susceptibility to infection. In turn, participation in a marathon race may influence nocturnal autonomic modulation and disturb homeostasis. Ultramarathon races may have such a long duration resulting in sleep deprivation even for several days, where sleep duration would be quite below the physiological range. It seemed that for ultramarathons of short duration, continuous running and sleep deprivation were beneficial for performance, whereas for races longer than 200 miles it was necessary to develop sleep strategies to sustain performance.

## Author contributions

All authors listed have made a substantial, direct, and intellectual contribution to the work and approved it for publication.
